# Editorial: Immune thrombocytopenia (ITP)—diagnosis and treatment

**DOI:** 10.3389/fmed.2024.1385113

**Published:** 2024-03-05

**Authors:** Tomás José González-López

**Affiliations:** Department of Hematology, Hospital Universitario de Burgos, Burgos, Spain

**Keywords:** immune, thrombocytopenia, ITP, diagnosis, treatment

ITP is characterized by a reduced platelet count in peripheral blood caused by a dysregulated immune response against platelets, affecting both their production and destruction. Despite important discoveries, such as the role of T-cell compartment and myeloid-derived suppressor cells (MDSCs) as key contributors to the development of ITP, the diagnosis of this disease remains one of exclusion. In addition, the clinical course of ITP is variable from subject to subject, reflecting its complex underlying pathophysiology.

The main goal of ITP treatment is to control and prevent bleeding maintaining platelet counts at a safe level. A better understanding of the ITP pathophysiology has helped to develop new targeted therapies. However, many of the issues surrounding the current diagnosis and treatment of ITP remain challenges that need to be widely understood.

Here we provide a snapshot of several challenges related to the diagnosis of ITP, e.g., the role of the serial platelet level index as a predictor of bleeding and the often forgotten links of immune thrombocytopenia with fatty metabolism and gut microbiota. Two other articles below address aspects of the therapeutic approach to ITP that have not often been described and which, in certain patient populations, may certainly influence the chances of therapeutic success of different drugs. Firstly, the fact that eltrombopag, a thrombopoietin receptor agonist (TPO-RA) drug, interacts with calcium-rich foods raises the question of the relationship between intermittent fasting in Muslim countries during Ramadan season. Secondly, the co-occurrence of coronary heart disease and ITP has intrinsic characteristics that influence and modulate how the disease is treated in this context. New potential tools for ITP such as Eijao Siwu decoction, a COVID19 vaccine-induced thrombotic thrombocytopenia case and a rare case of drug-induced immune-mediated haemolytic anemia were also included in our Research Topic.

Wu H-CG. et al. developed a method for early prediction of pulmonary hemorrhage (PH) after Stenotrophomonas maltophilia (SM) respiratory infection in patients with platelet counts < 150 × 10^9^/L. Their study aimed to evaluate risk factors of PH and to standardize an index measuring serial platelet deficit to predict PH in patients with this respiratory infection. After a retrospective review of 1.039 patients with positive SM cultures, authors compared clinical characteristics and laboratory parameters of both PH and non-PH groups reporting now a new platelet dissimilarity index (d-index). The patients with PH had increased international normalized ratio (INR), lower platelet counts, and higher platelet d-index. As a conclusion, low platelet counts (< 50 × 10^9^/L) are better to predict PH in patients with hematology/oncology or liver disease while d-index is better in patients with sepsis/treatment and various other groups.

Yu et al. provide an interesting study to determine if gut microbiota, fatty metabolism, and cytokines were associated with immune thrombocytopenia (ITP). They enrolled 29 ITP patients and 33 healthy volunteers analyzing fecal bacterial with a 16S rRNA sequencing. Flow cytometry and liquid chromatography-mass spectrometry (LC-MS) were used to determine plasma cytokines and motabolites, respectively. Authors observed some bacterial enrichment with upregulated plasma levels of various cytokines in ITP. On the contrary, depletion of some bacteria were correlated with certain downregulated plasma levels of some metabolites also in ITP. Thus, gut microbiota participate in the pathogenesis of ITP through affecting cytokine secretion, interfering with fatty metabolism.

Yassin et al. suggest that, among TPO-RA drugs, eltrombopag is not the preferred choice for fasting ITP patients because drug-food interaction affects its bioavailability. In addition, the consequent dietary restriction creates a challenge for eltrombopag patients regarding adherence and quality of life. Thus, after being fasted for several hours (e.g., Ramadan fasting), they do not recommend to administer eltrombopag with the fast-break meal. They also advise the switching between TPO-RAs as a useful strategy to avoid some limitations associated to TPO-RA use. In case of eltrombopag use, this should be administered with a 4-h fasting window.

Rahhal et al. provide an extensive review of the coronary artery disease (CAD) management in the setting of ITP. This challenge between thrombosis and bleeding lacks satisfactory management evidence. Following a systematic review of the available literature, the authors found that revascularisation by percutaneous coronary intervention or coronary artery bypass surgery with concurrent administration of corticosteroids and/or immunoglobulins (IVIG) for ITP was possible, but carried a significant risk of bleeding and mortality.

Wang et al. here report the efficacy of Ejiao Siwu (EJSW) decoction, a part of the traditional Chinese medicine for treating chronic diseases such as ITP. This decoction is composed of five Chinese medicinal herbs but unfortunately the mechanisms and molecular targets of EJSW for the treatment of ITP are still unclear. Active compounds of EJSW were identified by high-performance liquid chromatography-diode array detector (HPLC-DAD) and high-performance liquid chromatography-mass spectrometry (HPLC-MS). Authors identified 14 compounds and 129 targets, and 1,726 ITP targets. Further studies are needed to identify which of these may be effective compounds and targets in ITP.

Ihnatko et al. reported the first known case of COVID-19 vaccine-induced thrombotic thrombocytopenia (VITT) in Slovakia. This rare complication of adenoviral vector vaccine administration is presented as thrombocytopenia and thrombotic manifestations in various sites.

Finally, Wu Y. et al. report a drug-induced immune haemolytic anemia caused by cefoperazone-tazobactam/sulbactam combination therapy. Immune haemolytic anemia shares pathophysiological characteristics with ITP. Here authors found cefoperazone-dependent antibodies that activated complement and subsequently led to a drug-induced immune haemolytic anemia (DIIHA). No serological evidence of this association has been reported to date.

The articles in this Research Topic describe different and often insufficiently known aspects of the disease. Further work following the findings described therein will certainly contribute to a better understanding of the diagnosis and treatment of ITP.

## Author contributions

TJG-L: Writing—review & editing, Writing—original draft, Visualization, Validation, Methodology, Conceptualization.

